# Measuring connectedness to nature in preschool children in an urban setting and its relation to psychological functioning

**DOI:** 10.1371/journal.pone.0207057

**Published:** 2018-11-29

**Authors:** Tanja Sobko, Zhenzhen Jia, Gavin Brown

**Affiliations:** 1 School of Biological Sciences, Faculty of Science, The University of Hong Kong, Hong Kong; 2 The University of Hong Kong, Hong Kong, Hong Kong; 3 Faculty of Education & Social Work, The University of Auckland, Auckland, New Zealand; Institute of Physiology and Basic Medicine, RUSSIAN FEDERATION

## Abstract

**Background:**

The urban environment has been criticized for promoting ‘nature-deficit’ and ‘child-nature disconnectedness’. Keeping in mind the importance of nature exposure and its extensive health benefits, many environmental programs around the world hope to (re)connect children with nature. To evaluate the effectiveness of such efforts, valid tools to measure Connectedness to Nature (CN) are needed but do not exist today, especially for use with pre-schoolers.

**Methods:**

The original CN Index was modified and tested among the Parents of Preschool Children (CNI-PPC) in an urban setting (Hong Kong) for its internal consistency (*n* = 299) and external validity (*n* = 194). The ‘Strength and Difficulties Questionnaire’ (SDQ) was chosen for divergent and convergent analysis. Conventional recommendations for test adaptation and translation were used.

**Results:**

Confirmatory factor analysis (CFA) revealed that the 16-item scale adequately captured four major dimensions: enjoyment of nature, empathy for nature, responsibility toward nature, and awareness of nature (Cronbach’s α were respectively .86, .87, .75 and .80). When tested against the SDQ, a validated measure for child psychological functioning, and identification of children’s problem behaviours, three CNI-PPC factors influenced the SDQ outcomes: (1) the more enjoyment of nature children displayed the less overall distress and impairment they exhibited (β = -.64); (2) greater responsibility toward nature in children was associated with less hyperactivity (β = -.50), fewer behavioural and peer difficulties (β = -.62 and β = -.65 respectively) and improved prosocial behaviour (β = .77); (3) the more aware children were of nature, the less emotional difficulties they exhibited (β = -.51). The variance explained was large (range *R*^2^ = .42 to .80).

**Conclusions:**

Thus, CNI-PPC factors have meaningful and substantive associations with the strengths and difficulties parents perceive in their children. This indicates that the CNI-PPC is a valid and reliable instrument to measure CN at an age when children cannot respond for themselves. Further, this simple tool can help researchers/practitioners to better understand how connectedness to nature affects child psychological functioning and wellbeing.

**Trial registration:**

ClinicalTrials.gov Identifier: NCT02715544. Registered 8 March 2016.

## Background

Around 50 per cent of people worldwide, and 100 per cent of people in Hong Kong, live in an urban setting [1]. At the same time, around 78 per cent of urban Hong Kong is surrounded by open green areas and 90 per cent of the population lives within 400 metres of such areas [2]. This is comparable to the 300-meter indicator laid-out by the WHO for monitoring implementation of the Parma Declaration commitment to providing every child with access to “green spaces to play and undertake physical activity” [3, 4]. However, despite Hong Kong’s extensive, adjacent greenness, 16 per cent of pre-schoolers in Hong Kong and up to 22% in China show signs of mental health problems [5, 6].

Connectedness to Nature (CN) is defined as “an individual’s affective, experiential connection to nature” [7]. In adults, CN appears to consist of three components: cognitive (personal awareness or understanding of being a part of nature); affective (sensitivity towards nature protection); and behavioural (individual engagement in nature protection) [8]. In young children, CN has been suggested to be associated with two components (i.e., the cognitive and the affective) [9]. A ‘unique affinity’ for the natural environment, ‘ecological self’, and self-cognition related to the surrounding nature progresses from early childhood through adulthood [10]. Environmental attitudes develop early in life [11, 12] and early childhood appears to be a crucial period for developing a bond with nature [13]. During this developmental period, some important milestones relevant to CN (esp. *vis-à-vis* animal life) occur, including theory of mind, empathy, and affective perspective taking [14]. Around the age of two, children start responding to and caring for objects in the natural world [15] and by the time they enter school, children start to demonstrate a relationship with nature in multiple ways (e.g., trust vs. mistrust; attempting to make sense of what they see, hear, touch, taste, and smell; spatial autonomy with interest in micro-spaces, inside bushes, or under trees) [16–22]. Deeper understanding of preschoolers’ relationship with the natural world is warranted but remains difficult to achieve due to the lack of valid measuring tools, making it a significant knowledge gap.

### 1.1 Impact of connectedness to nature on health

Leisure activities of young children living in urban settings today have shifted from active, outdoor, nature-based play to passive indoor activities [23]. This shift has had negative impacts on children’s language and cognitive development and has even been associated with low self-confidence [24]. In contrast, contact with nature is positively associated with children’s length and quality of sleep [25], levels of physical activity [26], lower stress [27], superior cognitive skills (e.g., attention ability, working memory, intellectual and problem solving skills and creativity) [28–31]. Meanwhile, lower physical activity and higher sedentary behaviour have been associated with lower psychological well-being (reflected by total difficulties scores, peer problems, and emotional symptoms) as measured by the Strengths and Difficulties Questionnaire (SDQ), a validated measure for child psychological functioning [32]. In summary, exposure to nature contributes to both better physiological and mental health outcomes for children [33].

Keeping in mind the importance of exposure to nature and its health benefits, many environmental programs around the world, including in China, hope to (re)connect children with nature [34]. However, to evaluate the effectiveness of such efforts, robust tools to measure CN among all targeted populations are needed. At present, none of the existing methods for measuring children’s CN have been validated for use among preschool-aged children. Since children in this age group are at a critical point in becoming connected to nature, it is imperative that tools are available to capture this information. Hence, this study has tried to develop a tool that could reliably measure the degree of connectedness that young children have to their natural surroundings. The availability of such tools would help with evaluation of interventions, systems, or policies that seek to overcome barriers created by an urban environment.

### 1.2 Challenges in measuring connectedness to nature

Tools that measure CN are highly age-specific, partly because of the difficulty in separating emotional affinity toward nature (affective) from interest in nature (cognition) [35]. Among a growing number of CN measurement tools for adults, two are commonly used: the Nature Relatedness Scale (NRS) [36] and the Connectedness to Nature Scale (CNS) [7]. The Inclusion of Nature in Self (INS) [8] for school children uses single items and cannot be assessed for reliability. The only tool designed for younger school children, the Connection to Nature Index (CNI), targets 4th-grade students and includes items relating to children's feelings when in nature, their perceptions of human–nature relationships, and their concern for plants and animals; the themes were based on factors used in measurement tools for adults: (a) enjoyment of nature, (b) empathy for its creatures, (c) sense of oneness, and (d) sense of responsibility [37].

A number of challenges exist in investigating CN among preschool children. Clearly, their ability to make fine distinctions, especially between emotional affinity towards nature and cognitive interest, can be limited [7, 38]. Furthermore, younger children may have difficulties in distinguishing gradations in rating scales because they tend to think dichotomously (e.g., children are either awake or asleep) [39]. Nonetheless, it is highly likely that they have developed a degree of CN through their childhood [12]. Given that the 24-item CNI [37] was used with children aged 8–10 years old, it was the primary choice for adaptation for urban pre-schoolers. Still, the targeted group of this study differed from Cheng and Monroe’s (2012), who lived in an area outside major urban centres. The children in our sample were not only much younger (most did not attend school), but also lived in a highly urbanized context. Thus, a broad adaptation of the existing CNI was necessary. Furthermore, we explored the possibility of parents providing information about their children’s CN because parental-proxy measures of children’s activities, behaviours, emotions, and feelings have been widely used in early childhood research [40].

Two studies are reported. Study 1 reports the adaptation of the existing CNI into Chinese and pilot testing of the translated version with a sample of urban children and their parents; the Hong Kong setting presented a good example of such. Study 1 indicated the need to explore, through qualitative interviews, parental perceptions of their children’s experience of nature. This led to a revised pool of items for the Connectedness to Nature Index for Parents of Preschool Children (CNI-PPC). Study 2 used confirmatory factor analysis (CFA) to determine the degree to which the four-factor structure of the CNI-PPC could be recovered. Finally, the adjusted CNI-PPC was tested against the SDQ, a validated measure for child psychological functioning [32].

The CNI-PPC may be the first tool for understanding and predicting environmental attitudes and behaviours among preschool-aged children. The study was approved by the Ethical Committee of the lead author’s University.

## 2. Study 1. Piloting of the original CNI and its adaptations

This study aimed to (1) evaluate if the original CNI [37] was applicable for parents of pre-schoolers in a big city and (2) identify possible adaptations required to create a pool of valid items relating to the CNI factors. To ensure adherence to the principles of evaluation and adaptation of the existing CNI scale (i.e., familiarity of item contents, linguistic and cultural consistency, and systematic evidence approach), conventional recommendations for test adaptation and translation were used [41].

### 2.1 Methods

#### Stage 1

The original CNI items were translated/back-translated into Cantonese using traditional Chinese script. The randomly chosen parents of 31 children aged 2 to 5 (mean age = 2.16, *SD* = .90), together with their children, answered the 24 items, giving a rating for their child. The families were recruited from all over Hong Kong and lived in apartment buildings, without any access to front or back yards, which is typical of Hong Kong’s compact living environment. The inclusion criteria for participation in this study was that parents could understand and respond to the Cantonese version of the questions. The children, as reported by the parents, were physically and psychologically quite disconnected from nature and almost 50% of the original CNI items were marked as non-applicable by many parents. This indicated that families were relatively unable to meaningfully answer a high proportion of the items, raising doubts about the suitability of the original questionnaire for either the age group or the urban context.

#### Stage 2

To better understand the challenges reported by the participants responding to these original questions, 20 parents participated in either face-to-face or phone interviews about the CNI items. The semi-structured interview guide focused on environmental behaviours and was aligned with the four factors of the original CNI. The parents were asked if their children, despite living in a big city, already possessed certain feelings about nature and their reactions toward nature. They were encouraged to express opinions on the thematic issues around the questions, using their own words, and/or to suggest other wordings. Substitute items were generated in conversations with participants and finalised after all interviews were concluded. The audio records of the interviews were transcribed and subjected to thematic content analysis, using ThematiCoder [42].

#### Ethics approval and consent to participate

The study was approved by the Ethical Committee of the University of Hong Kong (nr EA1502073).

### 2.2. Results

The participants were able to comment on all four themes of the original CNI and rate their child on around half the items. Among those items (e.g., ‘When my child feels sad, he/ she likes to go outside and enjoy nature’), low mean scores were frequently reported. Interviews indicated that the low score arose because in an urban city like Hong Kong, going outside meant being in a built environment, rather than a natural one. Items, such as hearing the different sounds of nature, were rejected because of difficulties in hearing nature in the noisy, traffic-filled context of any big city.

The parents reported that the factor ‘Sense of Oneness’ was difficult to understand. Subsequent renaming of it as ‘Awareness of Nature’ allowed parents to understand it more easily. Moreover, the experience of being alone in nature, fundamental to the concept of ‘oneness with nature’ is a relatively rare experience for urban residents. On the other hand, expressing interest in natural objects (i.e., plants and animals) through books or being aware of their existence are relatively simple processes to initiate. This meant that awareness of nature was prioritised over an ineffable sense of being part of nature.

All but one of the original items belonging to ‘Sense of Responsibility’, except for one, had to be deleted. These items were complicated for participating families because urban residents have very little control over parks and gardens, which are usually controlled by government departments, and, as such, taking responsibility for the natural world is normally not exercised. The retained item had to do with littering, which is prohibited in big cities like Hong Kong. In connection to this, and based on feedback from the parents, an item on recycling was added. It is important to add that, when answering the questions, the parents were instructed to keep in mind the concept of responsibility toward nature in particular.

Moreover, parents considered the items from the ‘Empathy for Creatures’ factor to be too abstract, largely because most urban apartment dwellers manage their daily lives without exposure to wild animals. At the same time, ‘wild’ animals in Hong Kong can include monkeys and snakes, which are a hazard to society and, thus, elicit little empathy from residents. Three of the revised factor items focused instead upon care for living creatures and nature, which could include other families’ pets, stray animals in the street, and the extensive insect and bird life of the city. Indeed, care for caged crickets and birds is a classic Chinese practice, which is still seen in the older and residential streets of Hong Kong. The lack of answers for items of this factor suggests that respondents’ attitudes toward nature were shaped by the specific characteristics of an urban society. It, therefore, was not surprising that many of the assumptions underlying the original CNI items (developed in a more rural setting) were not applicable in a big city.

All the items were further adjusted to include the phrase ‘my child’ to ensure that the parents focused clearly on their own family’s experiences rather than those generally in Hong Kong. At the conclusion of this two-stage pilot process, a 20-item questionnaire (CNI-PPC) was developed and administered to a sample of Hong Kong parents of preschool-aged children.

## 3. Study 2. Large-scale evaluation

Firstly, the factor structure and criteria validity of the CNI-PPC were examined. Secondly, external validation of the CNI-PPC was performed, using the SDQ for divergent and convergent analysis. The SDQ was chosen on the presumption that a child’s problem behaviour would be negatively related to his/ her connectedness to nature.

The version of the SDQ used in this study has been validated in Hong Kong already, and its structure, internal consistency, and reliability was similar to the UK version[43]. It is a 25-item screening questionnaire that assesses preschoolers’ mental health over the past six months by five subscales of five items: emotional problems (symptoms), conduct problems, hyperactivity (inattention), peer relationship problems, and prosocial behaviors.

### 3.1 Methods

Families (*n* = 299) with children between 2 and 5 years of age (51% boys, mean age = 2.7, *SD* = 1.2) were recruited. All the parents (90.5% mothers, 9.1% fathers, and 0.3% others) were asked to complete the CNI-PPC, using a planned missing-data design, while two-thirds completed the SDQ (*n* = 194) about their child via the online survey software, SurveyMonkey. The survey links were sent to kindergartens in Hong Kong and to directly recruited parents. Online surveys have been found to yield more honest responses, save more time and cost compared to traditional paper surveys, especially in health fields [44, 45]. Responses to all the items were expressed on a 5-point balanced agreement scale (i.e., 1 = strongly disagree and 5 = strongly agree).

Before the final analysis of the surevy results, missing-value analysis removed participants with >10% missing values and imputed values for the remaining missing using the expectation maximization procedure [46]. Confirmatory factor analysis (CFA) was used to test the fit of the items to the intended factor structures of the CNI-PPC and the SDQ. Testing the fit of that model directly was appropriate, rather than conducting exploratory factor analysis (EFA) which presumes the items had zero face validity for the sub-scales of the CNI-PPC and the SDQ [47, 48]. Unlike EFA, CFA forces all non-specified paths to be zero, thus simplifying the data into, in this case, four factors with no paths from items to factors for which they were not specified. CFA was extended to the SDQ to ensure that the results of previous studies were applicable in this sample of Cantonese-speaking parents in Hong Kong.

Inspection of modification indices was used to identify poorly fitting items (e.g., low-loading or strong cross-loading to other items or factors), while ensuring decisions were conceptually valid. The fit of a CFA to the source data was determined by multiple indices, using conventional standards for non-rejection: the ratio of χ^2^ to *df* should be statistically not significant (*p*>.05), the gamma hat should be >.90, the root mean square of approximation (RMSEA), and the standardized root mean residual (SRMR) should be < .08 [49, 50].

To examine the relationships between the CNI-PPC and the SDQ, a general causal model was proposed. Because the literature supports the notion that exposure to and connection to nature is beneficial to well-being, we presumed that parent scores for the CNI-PPC would be causally predictive of perceived strengths and difficulties. We hypothesised in general that greater connectedness to nature would predict fewer difficulties and more strengths. To test this, we used structural equation modelling (SEM) to test our hypothesis by introducing the two CFA structures into a simultaneous set of linear regressions [47]. Evaluation of an SEM follows the same principles outlined above for CFA.

### 3.2 Results

#### 3.2.1 CNI-PPC measurement model

The original model of four factors for the CNI-PPC with 20 items did not meet conventional standards for acceptable fit (χ^2^ = 535.66; *df* = 164; χ^2^/*df* = 3.27, *p* = .07; CFI = .88; gamma hat = .89; RMSEA = .087; SRMR = .067). Items were trimmed from the model sequentially to ensure a simple structure. Modification indices indicated that item 19 had strong paths to multiple items in all the other factors, item 14 had strong paths to items in Responsibility toward Nature, item 11 was correlated with item 10, and item 16 had strong residual correlations with item 15. After this trimming, 16 items in four factors (i.e., Enjoyment of Nature [ENN], Empathy for Nature [EMN], Responsibility toward Nature [RN], and Awareness of Nature [AN]) had acceptable to good fit to the data (χ^2^ = 214.08; *df* = 98; χ^2^/*df* = 2.18, *p* = .14; CFI = .95; gamma hat = .95; RMSEA = .063; SRMR = .046). These four factors with retained items captured much of the original CNI’s four factors (Table 1), albeit with more concrete and limited definitions in Empathy for Nature, Responsibility toward Nature, and Awareness of Nature, making it more concrete and valid for the heavily built environment of a big city.

**Table 1:** Connectedness to nature index—parents of preschool children (CNI-PPC) items and factor descriptive statistics.

Factors	Items	Initial Loading	Final loading	*M* (SD)
Enjoyment of Nature (ENN) [*M =* 3.84, *SD* = 0.72; α = .86]				
1	My child likes to hear different sounds in nature	.76	.80	4.03 (0.88)
2	My child likes to see wild flowers in nature	.77	.79	4.00 (0.91)
3	Being in the nature makes my child feel peaceful	.71	.72	3.63 (0.90)
4	My child likes to garden and plant	.71	71	3.47 (0.96)
5	My child enjoys collect rocks and shells	.62	.63	3.81 (1.08)
7	My child enjoys touching animals and plants	.69	.69	4.07 (0.95)
Empathy for Nature (EMN) [*M =* 3.59, *SD* = 0.82; α = .87]				
6	My child feels sad when wild animals are hurt	.79	.84	3.73 (0.93)
12	My child is distressed when he/she sees animals being hurt	.87	.90	3.71 (0.95)
13	My child is heartbroken when animals pass away	.82	.79	3.33 (0.88)
14	My child is unhappy when the plants are dying*	.66	—	3.12 (0.89)
Responsibility toward Nature (RN) [*M =* 3.28, *SD* = 0.72; α = .75]				
8	My child believes that picking up trash on the ground can help the nature	.75	.73	3.35 (0.90)
11	My child treats plants, animals and insects with care	.72	.79	3.64 (0.92)
15	My child has a sense of responsibility and care for plants and animals*	.72	.70	3.20 (0.83)
16	My child is careful with wasting water*	.56	—	2.81 (0.97)
17	My child enjoys recycling paper and bottles	.60	.54	3.28 (0.93)
Awareness of Nature (AN) [*M =* 4.00, *SD* = 0.69; α = .80]				
9	My child notices wildlife wherever he/she is	.69	.71	3.95 (0.93)
10	My child chooses to read about plants and animals	.61	.64	3.72 (0.92)
18	My child feels the difference between outdoor and indoor	.70	.72	4.21 (0.82)
20	My child hears birds and other sounds in the nature	.79	.80	4.11 (0.83)
19	My child reflects/ knows that the food comes from nature*	.55	—	3.41 (0.94)

Note. Items marked with * were deleted in the final version.

Consistent with the fit indices, the Cronbach α values for the CNI-PPC were sufficient (Table 1), with three factors having α > .80, suggesting that factors had internal consistency. The standardized effect sizes between the CNI-PPC factor means were large between AN and SR in favour of AN (|*d*| = 1.02) and between ENN and SR in favour of ENN (|*d*| = 0.78), moderate differences (.40<|*d|* < .60) existed between EMN and all other factors, while ENN and AN had a small difference (|*d|* = .23).

#### 3.2.2 SDQ Measurement model

The five-factor model of SDQ consisted of correlated constructs with each construct reflecting five items. Fit statistics for the original Goodman five-factor model were acceptable (χ^2^ = 509.29; *df* = 265; χ^2^/*df* = 1.92, *p* = .17; CFI = .89; gamma hat = .91; RMSEA = .07; SRMR = .063). However, removal of two items from Hyperactivity produced an alternative trimmed model with good fit (χ^2^ = 402.06; *df* = 220; χ^2^/*df* = 1.83, *p* = .18; CFI = .91; gamma hat = .93; RMSEA = .065; SRMR = .056). The trimmed model had superior fit relative to the original model by a large margin (ΔAIC = 116.338) indicating it was better to use this factor structure. All five subscales had acceptable alpha values ranging from .70≤ α ≤ .83 (Table 2).

**Table 2:** SDQ items and factor descriptive statistics.

Factors and Items		Standardised factor loading
Emotional symptoms [*M =* 2.73, *SD* = 2.58; α = .80]		
3	Headache	.67
8	Worries	.80
13	Unhappy	.79
16	Clingy	.52
24	Fears	.61
Conduct problems [*M =* 2.73, *SD* = 2.42; α = .75]		
5	Tempers	.63
7	Obedient	-.52
12	Fights	.71
18	Lies	.52
22	Steals	.72
Hyperactivity [*M =* 4.50, *SD* = 3.01; α = .81]		
2	Restless	.84
10	Fidgety	.68
15	Distractible	.78
Peer Problems [*M =* 2.73, *SD* = 2.23; α = .70]		
6	Solitary	.61
11	Good friend	-.62
14	Popular	-.71
19	Bullied	.56
23	Good with Adults	.36
Prosocial behaviour [*M =* 7.64, *SD* = 2.47; α = .83]		
1	Considerate	.74
4	Shares	.60
9	Helpful	.74
17	Kind to kids	.76
20	Volunteer to help	.64

It is noteworthy that the two deleted items (#21 and #25) both correctly had inverse loadings from the Hyperactivity factor (-.52 and -.66 respectively). However, these two items, perhaps by being expressed in a positive way, did not associate purely with the negative construct hyperactivity and violated simple structure expectations. This could be seen in the strong modification indices (MI) between item 21 and item 20 (MI = 17.05) within the prosocial factor and the residual of item 21 and that of the prosocial behaviour factor (MI = 18.56) and also between the residual of item 25 and the residual of item 14 (MI = 10.65) in the peer problems factor. The lack of a simple structure for these two items warranted their removal from further analysis.

Inter-correlations among the SDQ factors were generally moderate (|*M*| = .62; range: -.69 ≤ *r* ≤ .74). Mean scores for three of the difficulties were below the mid-point of the response scale, with the mean for hyperactivity quite close to the response scale maximum. Prosocial strength was rated considerably higher. This suggests the participating students were largely developing normally, rather than clinically problematic. A number of local Hong Kong studies using the SDQ exist, but some methodological difficulties restricted comparability: the mean age was usually higher, the assessors were not the parents of the children assessed, or the reliability estimates of sub-scales were not satisfactory, ranging from .41 to .77, [51]. Considering the above, we compared our results to the best matching studies [43, 52], and concluded that the mean scores of our samples were relatively similar, except for the prosocial behaviour, which was rated somewhat higher on our study (7.6 vs 6.7).

#### 3.2.3 CNI-PPC and SDQ structural model

The factor inter-correlations within and between the CNI-PPC and SDQ are displayed in Table 3. Following multi-trait, multi-method conventions, the within-construct values are displayed in bold, while the between-construct values are in italics. Within the SDQ, the prosocial behaviour factor was negatively correlated with the four other factors consistently, with an average inter-correlation of *r* = -.45. The other SDQ factors had moderately positive inter-correlations, with an average of *r* = .46. Thus, the scales functioned as relatively independent facets of strengths and difficulties. In contrast, the within-construct inter-correlations in the CNI-PPC were stronger, with an average of *r* = .68.

**Table 3:** Inter-correlations within and between the CNI-PPC and SDQ factors.

	SDQ					CNI-PPC			
	I.	II.	III.	IV.	V.	VI.	VII.	VIII.	IX
*Strengths and Difficulties Questionnaire*									
I. Prosocial behaviour	**—**								
II. Hyperactivity/ inattention	-0.41**	**—**							
III. Conduct problems	-.16^*^	.51**	**—**						
IV. Peer problems	-.55**	.21**	.40**	**—**					
V. Emotional problems	-.69**	.56**	.41**	.67**	**—**				
*Connectedness to Nature Index—Parents of Preschool Children*									
VI. Enjoyment of Nature	.58**	*-*.43**	*-*.26**	*-*.53**	*-*.57**	**—**	.61**	.64**	.73**
VII. Empathy for Nature	.69**	*-*.34**	*-*.*11*	*-*.41**	*-*.53**		**—**	.77**	.68**
VIII. Responsibility toward Nature	.68**	*-*.38**	*-*.*06*	*-*.43**	*-*.54**			**—**	.66**
IV. Awareness of Nature	.65**	*-*.44**	*-*.26**	*-*.51**	*-*.59**				**—**

Note.

* = *p* < .05

** = *p* < .01

In terms of between construct inter-correlations, the Prosocial behaviour had a moderate positive correlation with each of the CNI-PPC scales, with an average of *r* = .65. In contrast, the four negative aspects of SDQ had a weaker but negative inter-correlation with the CNI-PPC factors with an average of *r* = -.40. Together, these scale inter-correlation values suggest that each scale provided sufficiently unique information so as to be warranted as a separate construct, and that the prosocial strengths were positively correlated with connectedness to nature in contrast to the SDQ difficulties scales. This provides convergent and divergent validation of the CNI-PPC.

Finally, to more robustly test the relations between the CNI-PPC and the SDQ, full-information structural equation modelling was used. The literature reviewed has indicated that connectedness to nature is positively associated with well-being. Hence, without suggesting specific hypotheses about the relationship of the CNI-PPC factors to individual SDQ scales, we propose that greater CNI-PPC ratings are positive predictors of strengths and negative predictors of difficulties that parents report their children as having. Hence, the structural equation model positions CNI-PPC factors as predictors of SDQ factors.

Given the relatively small sample (*N* = 194), the ratio of cases to variables becomes important. Full-information modelling with all items for both the CNI-PPC and the SDQ inventories involved 41 manifest variables giving a ratio of 4.73 cases per variable. This is close to the recommended threshold of 5:1 [53]. Because the SDQ scale scores involve summing all the contributing items, it is feasible to reduce each scale to a parcelled variable that is the sum of items. This approach would reduce complexity of the model to 22 items and give a ratio of 8.82:1 cases to variables.

**Fig 1:**
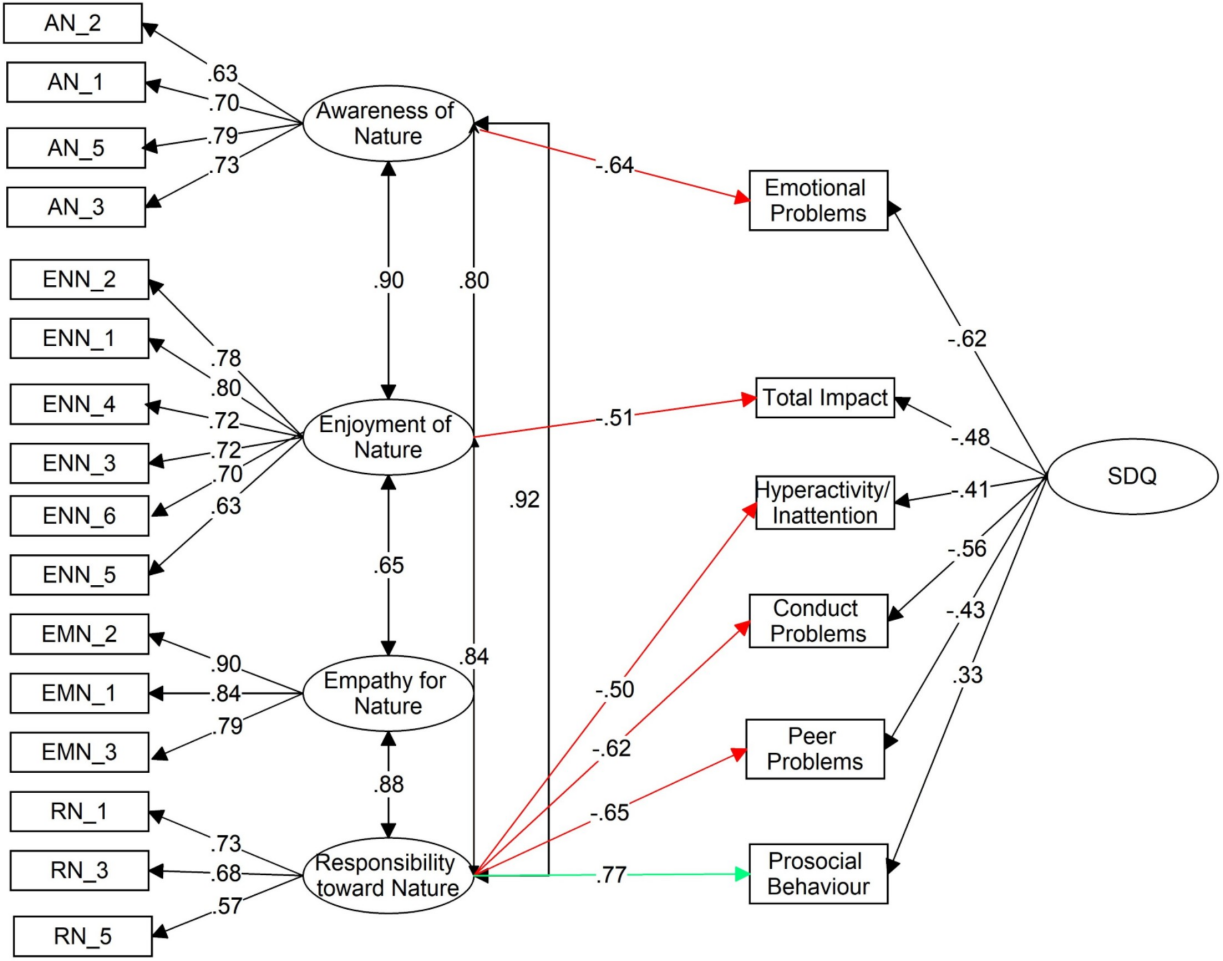
CNI-PPC as predictor of SDQ score. Note. Values are standardized; paths from CNI-PPC to SDQ scores shown as red when negative, green when positive; *R*^2^ values for SDQ scores displayed.

Full-information latent traits and items were used for the CNI-PPC, while the SDQ was modelled using factor-mean item parcels for each of the five SDQ scales and the total difficulties score. To capture the inter-correlated nature of the SDQ scales, a superordinate latent trait (SDQ) was introduced with a seed value placed on the prosocial strengths scale. Thus, the overall SDQ latent factor had negative paths to all SDQ difficulties scale scores except for prosocial behaviour. After removing all statistically non-significant paths, a well-fitting model was found (χ^2^ = 333.59; *df* = 197; χ^2^/*df* = 1.69 (*p* = .19); CFI = .95; Gamma hat = .94; RMSEA = .060 (90%CI = .049-.072; SRMR = .051) (Fig 1). Note that one item from the responsibility toward nature scale was deleted because of its strong inter-correlation with another item within the same scale; this deletion improved fit considerably (ΔAIC = 50.90).

All five of the SDQ scales were predicted by one of the four inter-correlated CNI-PPC factors. All, but Empathy for nature, of the CNI-PPC scales contributed to a more positive parental description of their child’s strengths and difficulties (Table 4). All CNI-PPC factors, except Empathy for Nature, predicted fewer difficulties, while only Responsibility toward Nature predicted increases in the strength of Prosocial Behaviour. Responsibility toward Nature contributed strongly to decreasing Peer Problems, Conduct Problems, and Hyperactivity/Inattention, while Enjoyment of Nature just contributed to a reduced total number of difficulties, and Awareness of Nature reduced Emotional Problems. In all relationships, the CNI-PPC had a stronger loading on the SDQ scale than the latent trait SDQ itself. The proportion of variance explained by the two predictors for each of the SDQ scales was large. Together these paths suggest that the CNI is a meaningful predictor of parental perceptions of the child’s strengths and difficulties [54]. This suggests overall that the greater a child’s connectedness to nature was perceived by their parent, the healthier the parent perceived the child being.

**Table 4:** Statistically significant paths from CNI-PPC to SDQ.

CNI-PPC Predictor		SDQ Dependency	CNI-PPC	SDQ	Total	Effect size
			β	β	*R*^*2*^	*f*^*2*^
Enjoyment of Nature		Impact Score	-0.64	-0.62	.80	4.00
Awareness of Nature		Emotional problems	-0.51	-0.48	.50	1.00
Responsibility toward Nature	{	Hyperactivity	-0.50	-0.41	.42	0.72
		Conduct problems	-0.62	-0.56	.70	2.33
		Peer problems	-0.65	-0.43	.62	1.63
		Prosocial Behaviour	0.77	0.33	.70	2.33

## 4. Discussion

The aim of this study was to adapt and validate a multi-dimensional instrument for measuring connectedness to nature in preschool children, living in a big city. The CNI-PPC was adapted and validated in a parent-proxy study. The resulting 16-item scale is the first tool to measure nature related attitudes and awareness for such a young population in the highly urbanised context of a major Asian city. It captures CN across four major dimensions, including Enjoyment of Nature, Empathy for Nature, Responsibility toward Nature and Awareness of Nature, which demonstrates its reliability. Results suggest that child’s relationship to nature, through parental perceptions, is most strongly characterized by Empathy for Nature, Enjoyment of Nature, Awareness of Nature, and Responsibility toward Nature.

As shown in Table 3 and Fig 1, the CNI-PPC factor inter-correlations ranged from moderate to high. This could be used to argue for treating the CNI-PPC as a single aggregate score rather than as a multi-dimensional construct. However, the current results suggest that there is more meaning to be found in allowing the separate factors to play a unique role. The paths shown in Fig 1 clearly indicate that Responsibility toward Nature is the most influential aspect of the CNI, with an almost universal prediction on all aspects of the SDQ. However, it is clear that Enjoyment and Awareness of Nature have unique relations to the SDQ despite their correlation with Responsibility toward Nature. Hence, we conclude at this stage of research, it makes more sense to retain the separate factors.

The CNI-PPC tool described here may give a unique opportunity to measure and further understand the relationship between connecting young children with nature and their psychological wellbeing. It is short, measures both affective and cognitive aspects of CN, and is based on the children’s personal experiences (via parental-proxy) of nature in a big city.

### 4.1 Comparison of CNI-PPC to CNI

Nonetheless, the new factors do not perfectly correspond to the factors of the original CNI. The differences appear to logically arise from the urban context of a big city and the younger age of the target population. The new Enjoyment of Nature factor excludes the quasi-therapeutic function of going into nature to combat sadness, but retains the original ideas of gaining joy by interacting with living things. The new Empathy for Nature retains strong elements of emotional attachment to living things, but removes the personal sense of unity with nature. The new Awareness of Nature has none of the transcendental unity implied by ‘sense of oneness’, rather it instead focuses on noticing and paying attention to living things. This recasting of “Oneness” as “Awareness” de-emphasises the possibility that any sense of self might be involved in CN. This result is in accordance with Cheng and Monroe (2012), who pointed out the difficulty of composing ‘self in nature’ items for nine-year olds [37].

Lastly, the Responsibility toward Nature factor restricts action to littering and tidiness, rather than to making a difference to the natural world. This corresponds with the reflection that very little is known about the concept of responsibility in young children, as children lack the competence and experience to be responsible [55]. At the same time, Bjerke argues that responsibility is not a static entity, ‘but a complex and rich practice embedded in relationship with others in the sense that it is judged in relation to the actions and attitudes of others’ [55]. In his comprehensive study, three categories of responsibilities are described: personal responsibility, social responsibility, and collective responsibility, all of which may be seen to be reflected in our items. Deducing from the qualitative analysis of the individual interviews with children, Bjerke suggests that “children will only learn to become responsible if they are given opportunities to acquire experience and practice to be responsible” (p.73) [55] and are, therefore, in need of practice. Interestingly, the notion of personal responsibility seems to be linked to children’s prosocial behaviour already in preschool and early school years, which is one of the findings of external validations of the CNI [56]. However, the factor Responsibility toward Nature is likely to be susceptible to social desirability bias in a parent-report survey and we would like the readers and future users to be aware of this.

### 4.2 Convergent validity of the CNI-PPC

Our studies have confirmed the expectation that CN would be related to psychological functioning and parental perceptions of children’s social and emotional well-being. This corresponds with two recent reviews showing that outdoor nature spaces and activities interacting with nature benefit both mental and physical health of human beings [57, 58]. In our study, greater endorsement of the child’s enjoyment of, awareness of, and responsibility toward nature resulted in reduced perceived behavioural difficulties. Finally, the more parents rated their child as responsible for nature, the less hyperactivity, conduct, and peer problems were perceived.

These relationships are entirely consistent with studies that have shown there is an association between: (a) urban green space exposure and increased physical and emotional wellbeing [59, 60], (b) a lack of urban green spaces in a child's neighbourhood and increased mental health and behavioural problems [33, 61], including increasing distance to urban green spaces with greater hyperactivity/inattention and peer relationship problems, with the effect being strongest for children residing in the inner city [62], (c) the use of green spaces and residential surrounding greenness with fewer behavioural problems (similarly measured with the SDQ) [61], and (d) a connectedness to nature score and greater life satisfaction and overall happiness [7]. Clearly, greater access to nature is a challenge for families with younger children in any urban setting. In such settings, where there may be relatively good access to minor green spaces near residences, but relatively weak traditions of taking children into major green spaces in the suburbs or outlying regions.

### 4.3 Significance of CNI-PPC

We believe this is the first instrument to measure CN at such a young age, which makes it unique. Monitoring of child’s behaviour and early psychological development has a crucial role in paediatric care [63], because early identification of difficulties and intervention may influence further reduction of difficulties and improve the quality of family lives [64, 65].

Measuring the relationship between individuals and the natural world is challenging, even with adults. Measuring relationships with nature among pre-schoolers is even more complex, requiring a different tool and methodology from those previously developed. Ideally, an instrument would be simple enough for children who cannot read or write; however, this is unlikely to be achieved for such children. Fortunately, parents are normally literate and are in a position to give a good estimate of their child’s CN. Thus, assessment of CN among pre-school children may be only possible through a child’s parents. To our knowledge, none of the existing tools for measuring CN have been applied to pre-schoolers, the age when we believe it is necessary to establish a baseline for any health-promoting interventions. We expect that this instrument will be sensitive to changes over time, which will be one of the major advantages of its application. Ultimately, the goal is to measure changes during any nature promoting interventions early in life, including how such changes affect lifestyle habits and health in general. The knowledge gained from these investigations will inform future practice and provide valuable information on the best approaches to promote CN in children from preschool onwards. Hence, the tool validated here fills the existing methodological gap, providing a simple possibility to assess CN in young children in an urban population.

### 4.4 Future research

There are several limitations to the present study. Firstly, because of its exploratory design, the causal modeling reported here cannot be proven because the data are correlational. However, the model is based on a presumed directionality of relationship; that is, the modelling approach assumes causality and models the relationships as causal [66]. Successful modelling, thus, forms the grounds for future experimental studies that could prove the hypothesised relations seen in this study. Hence, to explore this, we are currently conducting a randomised, controlled trial (RCT), collecting data objectively by measuring a number of health-related outcomes crossed with location information (i.e., urban green spaces or otherwise). Furthermore, given the restriction of constructs in the model, it is not possible to determine whether the observed effects might have arisen from a lagged association between CN and parents’ perceived difficulties in children or some other influential variable such as socio-economic status or a child’s individual conscientiousness. Additonally, it was not possible to control for parental psychopathology, parenting styles, or parental CN and these, in turn, may have influenced the results. Nonetheless, the obtained sample size (*n* = 299) was large enough to detect linear bivariate regressions of β = .20 with substantial power (α = .05; 1-β = .94) [67]. Hence, the current results are not likely to be inaccurate estimates of population parameters. Social desirability may inflate relationships between nature connectedness and psychological health. Future research could examine the specific relationship between nature-related enjoyment, empathy, and so on with psychological health, controlling for non-nature-related enjoyment, empathy, et cetera. Finally, if interaction with nature is shown to have associations with cognitive and affective outcomes, a potential benefit of a CN tool for young children is the ability to test if interactions with nature are more/less beneficial for children with high/low CN. This would support the creation of customised health promotion or landscape design interventions.

To estimate the practical research value of a new tool, an assessment of its use in other urban settings should be undertaken. A further application of the tool would be to explore and compare urban, suburban, and rural children including differences in how they perceive and appreciate the natural environment surrounding them. A specific next step would be to investigate differences in the CN between children living in the inner city of Hong Kong compared to its surrounding areas. Such a study would build upon recent findings about the positive effect of green spaces on the mental health of children being different between those living in the outskirts of a city compared to those living in the inner city, as well as the role played by the types of green spaces (open countryside, smaller parks) available to them [62]. Many inner cities lack large open green spaces, and smaller parks/gardens would be expected to play a more important role for urban families than for children living in the outskirts or rural areas, where bigger green spaces are more prevalent.

The lack of CN measurement instruments for children of a young age does not allow us to answer very important questions such as: Has psychological functioning in Hong Kong deteriorated over time in relation to disconnection from nature? Since when have children in Hong Kong lived with little connection to nature? What type of ‘nature’ do Hong Kong children wish/need to connect with? How has the loss of CN affected cultural and traditional practices related to psychological functioning? Finding answers to these questions may elucidate some plausible connections concering the psychological functioning of children in big cities like Hong Kong.

It must also be noted that perceptions of CN seem to vary not only with age but also with cultural context [68]. The present version of the CNI-PPC was developed for Hong Kong pre-schoolers, though it could be potentially useful for children in other Chinese-speaking communities (e.g., Macau, Taiwan, Guangzhou, etc.) and other urban settings worldwide. Being a parental-proxy tool, the language may not be a barrier, and this short questionnaire could potentially be adapted for use for non-Chinese-speaking children in other communities. However, more research is needed to examine the psychometric properties of the tools in Chinese-speaking and English-speaking sample populations. The reliability of the culturally dependent sub-scales should be further tested. Furthermore, cut-off scores should be developed, tested, and standardized taking into account relevant demographic characteristics that might be associated with SDQ scores.

## 5. Conclusion

To conclude, the CNI-PPI has proved to be a satisfactory psychometric tool in regard to its internal consistency and its logically coherent convergence to and divergence from the dimensions of the SDQ (i.e., positive to prosocial and negative to all other difficulty aspects). It is likely to be a useful screening tool for paediatricians and early childhood educators in the identification of children who may need further help in their developmental status. Being a short and user-friendly tool, it could also be a useful research tool for researchers working on child cognitive development. It could potentially be used as a tool for the evaluation of early childhood intervention programmes designed to enhance children’s early development and well-being.

## Abbreviations

AN: Awareness of Nature
CFA: Confirmatory factor analysis
CN: Connectedness to Nature
CNI-PPC: Parents of Preschool Children
CNS: Connectedness to Nature Scale
EFA: exploratory factor analysis
SDQ: Strength and Difficulties Questionnaire
ENN: Enjoyment of Nature
INS: Inclusion of Nature in Self
NRS: Nature Relatedness scale
RCT: randomised controlled trial
RMSEA: root mean square of approximation
RN: Responsibility toward Nature
